# Real-Time Multi-Target Localization from Unmanned Aerial Vehicles

**DOI:** 10.3390/s17010033

**Published:** 2016-12-25

**Authors:** Xuan Wang, Jinghong Liu, Qianfei Zhou

**Affiliations:** 1 Changchun Institute of Optics, Fine Mechanics and Physics, Chinese Academy of Sciences, Changchun 130033, China; liujinghong@ciomp.ac.cn (J.L.); zhouqianfei@ciomp.ac.cn (Q.Z.); 2 University of Chinese Academy of Sciences, Beijing 100049, China

**Keywords:** multi-target localization, UAV, real time, lens distortion correction, RLS

## Abstract

In order to improve the reconnaissance efficiency of unmanned aerial vehicle (UAV) electro-optical stabilized imaging systems, a real-time multi-target localization scheme based on an UAV electro-optical stabilized imaging system is proposed. First, a target location model is studied. Then, the geodetic coordinates of multi-targets are calculated using the homogeneous coordinate transformation. On the basis of this, two methods which can improve the accuracy of the multi-target localization are proposed: (1) the real-time zoom lens distortion correction method; (2) a recursive least squares (RLS) filtering method based on UAV dead reckoning. The multi-target localization error model is established using Monte Carlo theory. In an actual flight, the UAV flight altitude is 1140 m. The multi-target localization results are within the range of allowable error. After we use a lens distortion correction method in a single image, the circular error probability (CEP) of the multi-target localization is reduced by 7%, and 50 targets can be located at the same time. The RLS algorithm can adaptively estimate the location data based on multiple images. Compared with multi-target localization based on a single image, CEP of the multi-target localization using RLS is reduced by 25%. The proposed method can be implemented on a small circuit board to operate in real time. This research is expected to significantly benefit small UAVs which need multi-target geo-location functions.

## 1. Introduction

Real-time multi-target localization plays an essential and significant role in disaster emergency rescue, border security and so on. Over the past two decades, considerable research efforts have been devoted to multi-target localization. UAV electro-optical stabilized imaging systems are equipped with many kinds of sensors, including visible light cameras, infrared thermal imaging systems, laser range finders and angle sensors. Target localization needs to measure the attitude of the UAV, the attitude of the electro-optical stabilized imaging system and the distance between the electro-optical stabilized imaging system and the target.

The target localization methods from UAVs are divided into two categories. One category is target localization using a group of UAVs [1,2,3,4,5]. The other category is target localization using a single UAV [6,7,8,9,10]. This research aims to improve the effectiveness and efficiency of target localization from a single UAV. Particularly, this research proposes a new hybrid target localization scheme which integrates both zoom lens distortion correction and an RLS filtering method. The proposed scheme has many unique features which are designed to geo-locate targets rapidly.

Previous studies on geo-locating targets from a fixed-wing UAV have several limitations. Deming [1] described a probabilistic technique for performing multiple target detection and localization based on data from a swarm of flying optical sensors. Minaeian [2] described a vision-based crowd detection and Geographic Information System (GIS) localization algorithm for a cooperative team of one UAV and a number of unmanned ground vehicle (UGV)s. Morbidi [3] described an active target-tracking strategy to deploy a team of unmanned aerial vehicles along paths that minimize the uncertainty about the position of a moving target. Qu [4] described a multiple UAV cooperative localization method using azimuth angle information shared between the UAVs. Kwon [5] described a robust, improved mobile target localization method which incorporates the Out-Of-Order Sigma Point Kalman Filter (O3SPKF) technique.

In [1,2,3,4,5], target location methods based on data fusion technology have to use a group of UAVs. Target localization using a group of UAVs has some issues, including the high computational complexity of data association, complexity of UAV flight plans, difficulties in efficient data communication between UAVs and high maintenance costs due to the use of multiple UAVs. This paper presents a method for determining the location of objects using a gimbaled EO camera on-board a fixed-wing unmanned aerial vehicle (UAV). We focus on geo-locating targets using a single fixed-wing UAV due to the low maintenance costs. A single fixed-wing UAV (as opposed to rotary wing aircraft) has unique benefits including adaptability to adverse weather, good durability and high fuel efficiency.

In [6], Yan used absolute height above sea level of a UAV to geo-locate targets. In contrast, our system focuses on geo-locating targets in the video stream and does not require absolute height above sea level of the UAV data. In [7], Ha used scale invariant feature transform (SIFT) to extract feature points of the same target in different frames. The author calculated the relative height between the target and the UAV by three dimensional reconstruction. The location accuracy depends on the accuracy of this three dimensional reconstruction. The method thus requires a large amount of computation. In contrast, the accuracy of our system compared to that described in [7] is almost the same, but our system has a great advantage in reduced computational complexity, so our system can geo-locate 50 targets at the same time and improve the efficiency of multi-target localization. This is very important in military reconnaissance and disaster monitoring applications which require good real-time performance.

In [8], Barber introduced a system for vision-based target geo-localization from a fixed-wing micro-air vehicle. In flight tests, the UAV geo-locates the stationary target when the UAV orbits the targets. In [9], the UAV flies in an orbit in order to improve the geo-location accuracy. In [10], the authors assume that the UAV’s altitude above the target is known. The target’s altitude is obtained from a geo-referenced database made available by the Perspective View Nascent Technology (PVNT) method. In [11], target-location need an accurate geo-referenced terrain database. In [12], all information collected by an aerial camera is accurately geo-located through registration with pre-existing geo-reference imagery. In contrast, our system focuses on geo-locating a specific object in the video stream and does not require any preexisting geo-referenced imagery.

In all the above references, the UAVs are equipped with fixed-focal lenses. The authors do not take into account the effect of zoom lens distortion on multi-target localization. Many electro-optical stabilized imaging system are equipped with zoom lenses. The focal length of a zoom lens is adjusted to track targets at different distances during the flight. The zoom lens distortion varies with changing focal length. Real-time zoom lens distortion is impossible to correct by using calibration methods because the large amount of transformation calculation has to be repeated when the focal length is changed.

The primary contributions of this paper are: (1) the accuracy of multi-target localization has been improved due to the combination of a real-time zoom lens distortion correction method and a RLS filtering method using embedded hardware (a multi-target geo-location and tracking circuit board); (2) UAV geo-locates targets using embedded hardware (the multi-target geo-location and tracking circuit board) in real-time without orbiting the targets; (3) 50 targets can be located at the same time using only one UAV; (4) the UAV can geo-locate targets without any pre-existing geo-referenced imagery, or a terrain database; (5) the circuit board is small, and therefore, can be applied to many kinds of small UAVs; (6) multi-target localization and tracking techniques are combined, therefore, we can geo-locate multiple moving targets in real-time and obtain the target motion parameters such as velocity and trajectory. This is very important for UAVs performing reconnaissance and attack missions.

The rest of paper is organized as follows: Section 2 briefly presents the overall framework of the multi-target localization system. Section 3.1 presents the reference frames and transformations required for the multi-target localization system. Section 3.2 presents our multi-target geo-location model. Section 4 presents the methods to improve the accuracy of multi-target localization. Section 4.1 presents the distortion correction method. Section 4.2 presents the RLS filter method. Section 5 presents the results of multi-target localization for aerial imaged captured from a flight test and evaluates their accuracy. Section 6 presents the conclusions.

## 2. Overall Framework

**Figure 1:**
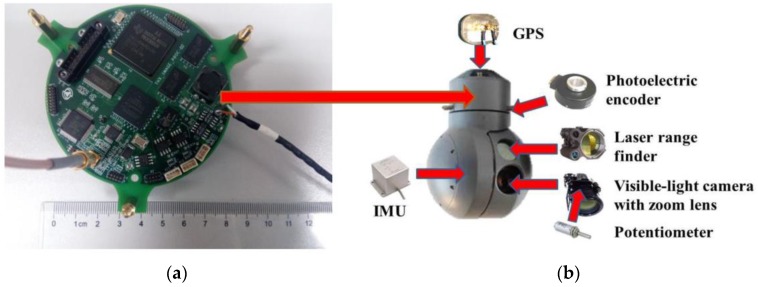
(**a**) Multi-target geo-location and tracking circuit board; (**b**) Electro-optical stabilized imaging system. The arrows in the Figure 1b represent the installation locations of main sensors in an electro-optical stabilized imaging system.

The real-time multi-target geo-location algorithm in this paper is programmed and implemented on a multi-target geo-location and tracking circuit board (model: THX-IMAGE-PROC-02, Changchun Institute of Optics, Fine Mechanics and Physics, Chinese Academy of Sciences, Changchun, China, see Figure 1a) with the TMS320DM642 (Texas Instruments Incorporated, Dallas, TX, USA) @ 720 MHz Clock Rate and 32 Bit Instructions/Cycle and 1 GB double data rate synchronous dynamic random access memory (DDR SDRAM). This circuit board also performs the proposed zoom lens distortion correction and the RLS filtering in real-time. The multi-target geo-location and tracking circuit board is mounted on an electro-optical stabilized imaging system (see Figure 1b). This aerial electro-optical stabilized imaging system consists of a visible-light camera, a laser range finder, an inertial measurement unit (IMU), a global positioning system (GPS), and a photoelectric encoder. They are in the same gimbal so that they rotate altogether in the same direction in any axis.

The electro-optical stabilized imaging system is mounted on the UAV (model: Changguang 1, Changchun Institute of Optics, Fine Mechanics and Physics, Chinese Academy of Sciences, Changchun, China) to stabilize the videos and any eliminate video jitters caused by the UAV therefore greatly reducing the impact of external factors.

**Figure 2:**
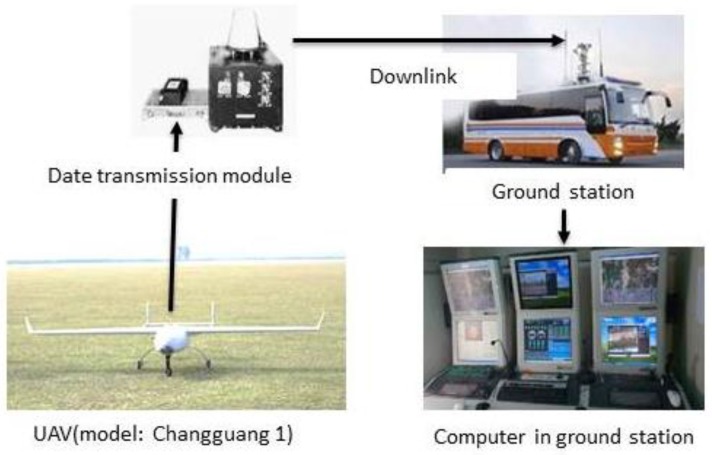
UAV system architecture.

The UAV system incorporates the electro-optical stabilized imaging system, UAV, data transmission module and ground station, which is shown in Figure 2. In the traditional target geo-location algorithms [1,2,3,4,5,6,7,8,9,10,11,12], the image and UAV attitude information are transmitted to a ground station. The target geo-location is calculated on a computer in the ground station. However, the date transmission model sends data with divided time mode, so the image and UAV attitude information are respectively transmitted at different times from the UAV to the ground station, so it is not guaranteed that the image and UAV attitude information will be obtained at the same time in ground station. Therefore, the traditional target geo-location algorithm on the computer in the ground station has poor real-time ability and unreliable target geo-location accuracy.

To overcome the shortcomings of traditional target geo-location algorithms such as algorithm complexity, unreliable geo-location accuracy and poor real-time ability, in this paper, the target geo-location algorithm is implemented on a multi-target geo-location and tracking circuit board on the UAV in real-time. Real-time ability is very important for urgent response in applications such as military reconnaissance and disaster monitoring.

**Figure 3:**
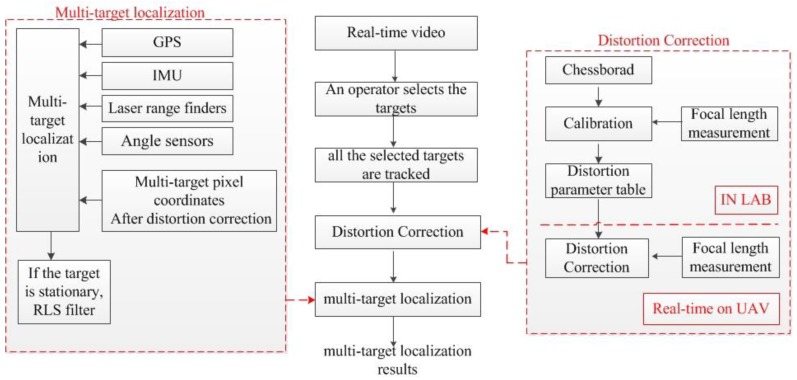
The overall framework of the multi-target geo-location method.

The overall framework of the multi-target geo-location method is shown in Figure 3. The detailed workflows of the abovementioned multi-target geo-location method will be introduced as follows: we use UAV to search for the ground targets, which are selected by an operator in the ground station. The coordinates of the multiple targets in the image are transmitted to the UAV through a data transmission model. Then, all the selected targets are tracked automatically by the multi-target geo-location and tracking circuit board using the improved tracking method based on [13]. The electro-optical stabilized imaging system locks the main target in the field of view (FOV) center. Other targets in the FOV are referred to as sub-targets. The electro-optical stabilized imaging system measures the distance between the main target and the UAV using a laser range finder.

In order to ensure that the image, UAV attitude information, electro-optical stabilized imaging system’s azimuth and elevation angle, laser range finder value, and camera focal length are obtained at the same time, the frame synchronization signal of the camera is used as the external trigger signal for data acquisition by the above sensors, so we don’t need to implement sensor data interpolation algorithms in the system except for the GPS data. The UAV coordinates interpolation algorithm is shown in Equations (35) and (36).

The multi-target geo-location and tracking circuit board computed the multi-target geo-location after lens distortion correction in real-time. Then, the board used the moving target detection algorithm [14,15,16,17,18] for the tracked targets. If the target which is tracked is stationary, the multi-target geo-location and tracking circuit board uses the RLS filter to improve the target geo-location accuracy. The multi-target geo-location results are superimposed on each frame in the UAV and downlinked to a portable image receiver and the ground station.

This research aims to address the issues of real-time multi-target localization in UAVs by developing a hybrid localization model. In detail, the proposed scheme integrates the following improvements:

(a) The multi-target localization accuracy is improved due to the combination of the zoom lens distortion correction method and the RLS filtering method. A real-time zoom lens distortion correction method is implemented on the circuit board in real time. In this paper, we analyse the effect of lens distortion on target geo-location accuracy. Many electro-optical stabilized imaging systems are equipped with zoom lenses. The focal length of a zoom lens can be adjusted to track targets at different distances during the flight . The zoom lens distortion varies with changing focal length. Real-time distortion correction of a zoomable lens is impossible by using the calibration methods because the tedious calibration process has to be repeated again if the focal length is changed.
(b) The target geo-location algorithm is implemented on a circuit board in real time. The size of the circuit board is very small, therefore, this circuit board can be applied to many kinds of small UAVs. The target geo-location algorithm has the following advantages: low computational complexity and good real-time performance. UAV can geo-locate targets without pre-existing geo-referenced imagery, terrain databases and the relative height between UAV and targets. UAV can geo-locate targets using the embedded hardware in real-time without orbiting the targets.
(c) The multi-target geo-location and tracking circuit board use the moving target detection algorithm [14,15,16,17,18] for the tracked targets. If the target which is tracked is stationary, the multi-target geo-location and tracking circuit board uses the RLS filter to automatically improve the target geo-location accuracy.
(d) The multi-target localization, target detection and tracking techniques are combined. Therefore, we can geo-locate multiple moving targets in real-time and obtain target motion parameters such as velocity and trajectory. This is very important for UAVs performing reconnaissance and attack missions.

The real output rate of the geo-location results is 25 Hz. The reasons are as follows:

(a) The data acquisition frequency of all the sensors is 25 Hz: the visible light camera’s frame rate is 25 Hz. The frame synchronization signal of the camera is used as the external trigger signal for all sensors expect GPS (the UAV coordinates interpolation algorithm is shown in Equations (35) and (36)).
(b) Lens distortion correction is implemented in real-time, and the output rate of target location results after the lens distortion correction is 25 Hz.
(c) When it is necessary to locate a new stationary target, the RLS algorithm needs 3–5 s to converge to a stable value (within 5 s, lens distortion correction is implemented in real-time, the output rate is 25 Hz). After 5 s, the geo-location errors of the target have converged to a stable value. We can obtain a more accurate location of this stationary target immediately (it is no longer necessary to run RLS). That output rate is 25 Hz, too.

Our geo-location algorithm can geo-locate at least 50 targets simultaneously. The reasons are as follows:

(a) For a moving target we only use lens distortion correction to improve the target geo-location accuracy. This consumes 0.4 ms on average when calculating the geo-location of a single target and, at the same time, correcting zoom lens distortion (Section 5.4). It consumes 0.4 ms for tracking the multiple targets (Section 3.3). The image frame rate is 25 fps, so the duration of a frame is 40 ms, so our geo-location algorithm can geo-locate at least 50 targets simultaneously.
(b) For a stationary target, only when it is necessary to locate a new stationary target, the RLS algorithm needs 3–5 s to converge to a stable value (within 5 s, lens distortion correction is implemented in real-time, 50 targets can be located simultaneously). After 5 s, the geo-location errors of the target have converged to a stable value. We no longer need to run RLS, so our geo-location algorithm can geo-locate at least 50 targets simultaneously after lens distortion correction and RLS.

Therefore, this algorithm has great advantages in geo-location accuracy and real-time performance. The multiple target location method in this paper can be widely applied in many areas such as UAVs and robots.

## 3. Real-Time Target Geo-Location and Tracking System

### 3.1. Coordinate Frames and Transformation

**Figure 4:**
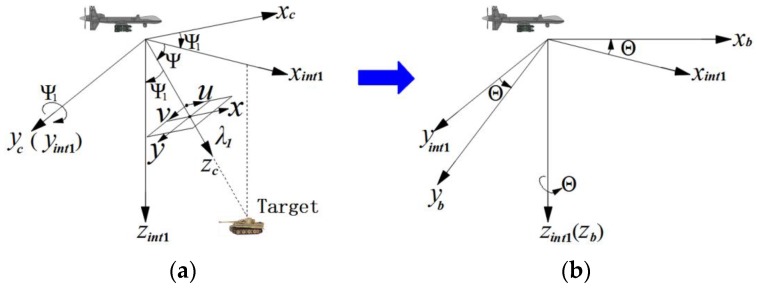
The coordinate frames relation: Azimuth-elevation rotation sequence between camera and body frames. (**a**) camera frame; (**b**) body frame.

Five coordinate frames (camera frame, body frame, vehicle frame, ECEF frame and geodetic frame) are used in this study. The relative relationships between the frames are shown in Figure 4. All coordinate frames follow a right-hand rule.

#### 3.1.1. Camera Frame

The origin is the camera projection center. The x-axis $x_{c}$ is parallel to the horizontal column pixels’ direction in the CCD sensor (i.e., the u direction in Figure 4). The y-axis $y_{c}$ is parallel to the vertical row pixels’ direction in the CCD sensor (i.e., the v direction in Figure 4). The positive z-axis $z_{c}$ represents the optical axis of the camera.

#### 3.1.2. Body Frame

The origin is the mass center of the attitude measuring system. The x-axis $x_{b}$ is the 0° direction of attitude measuring system. The y-axis $y_{b}$ is the 90° direction of attitude measuring system. The z-axis $z_{b}$ completes the right handed orthogonal axes set. The azimuth Θ, elevation angle ψ and distance $\lambda_{1}$ output by electro-optical stabilized imaging system are relative to this coordinate frame.

#### 3.1.3. Vehicle Frame v

A north-east-down (NED) coordinate frame. The origin is the mass center of attitude measuring system. The aircraft yaw β, pitch ε and roll angle γ output by the attitude measuring system are relative to this coordinate frame.

#### 3.1.4. ECEF Frame

The origin is Earth’s center of mass. The z-axis $z_{e}$ points to the Conventional Terrestrial Pole (CTP) defined by International Time Bureau (BIH) 1984.0, and the x-axis $x_{e}$ is directed to the intersection between prime meridian (defined in BIH1984.0) and CTP equator. The axes $y_{e}$ completes the right handed orthogonal axes set.

#### 3.1.5. WGS-84 Geodetic Frame

The origin and three axes are the same as in the ECEF. Geodetic longitude L, geodetic latitude M and geodetic height H are used here to describe spatial positions, and the aircraft coordinates $\left(L_{0},M_{0},H_{0}\right)$, output by GPS are relative to this coordinate frame.

The relation between camera frame and body frame is shown in Figure 4. Two steps are required. First, transformation from camera frame to intermediate frame int1: rotate 90° (elevation angle ψ) along the y-axis $y_{c}$. The next step is transformation from intermediate frame int1 to body frame: rotate azimuth angle Θ along the z-axis $z_{int1}$. In Figure 4a, $\psi_{1}$ represents 90°.

**Figure 5:**
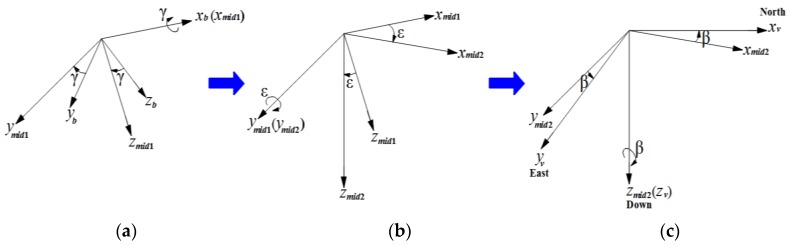
The coordinate frames relation: roll-pitch-yaw rotation sequence between body and vehicle frames. (**a**) body frame; (**b**) intermediate frame; (**c**) vehicle frame.

The relation between body frame and vehicle frame is shown in Figure 5. Three steps are required. First, transformation from the body frame to the intermediate frame mid1: rotate roll angle γ along the x-axis $x_{b}$. The next step is transformation from the intermediate frame mid1 to the intermediate frame mid2: rotate pitch angle ε along the y-axis $y_{mid1}$. The final step is transformation from the intermediate frame mid2 to the vehicle frame: rotate yaw angle β along the z-axis $z_{mid2}$.

**Figure 6:**
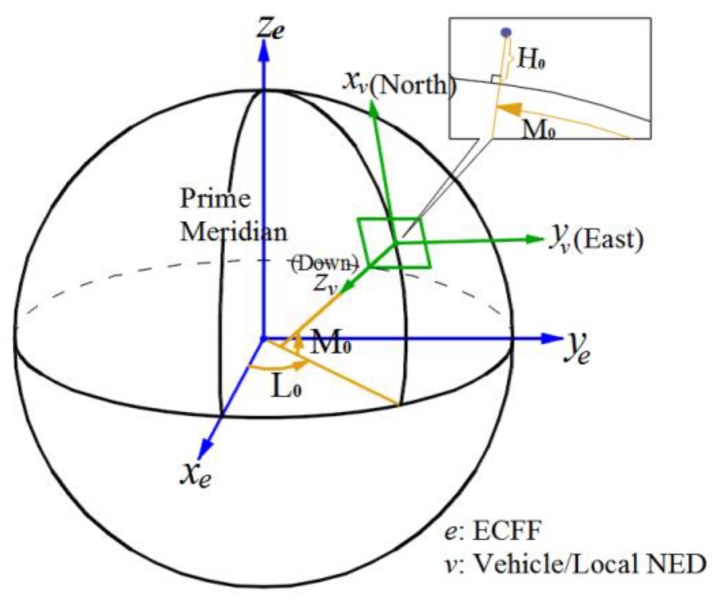
The coordinate frames relation: Vehicle, ECEF and geodetic frames.

The relation between vehicle frame and earth centered earth fixed (ECEF) is shown in Figure 6.

### 3.2. Multi-Target Geo-Location Model

**Figure 7:**
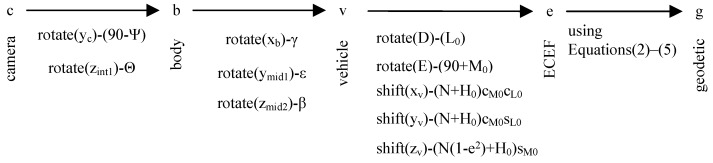
Coordinate transformation process of multi-target geo-location system.

As shown in Figure 4a, the main target is at the camera field of view (FOV) center, whose homogeneous coordinates in the camera frame are $\left[x_{c},y_{c},z_{c},1\right]{\;}^{T}=\left[0,\;0,\;\lambda_{1},1\right]$, Through the transformation among five coordinate frames ranging from camera frame to WGS-84 geodetic frame, the geographic coordinates of main target in the WGS-84 geodetic frame can be determined, as shown in Figure 7.

First, we calculate the coordinates of the main target in the ECEF:

(1) $$\begin{array}{l} \left[\begin{array}{c} x_{e}\\ y_{e}\\ z_{e}\\ 1 \end{array}\right]=\left[\begin{array}{cccc} -c_{L_{0}}s_{M_{0}} & -s_{L_{0}} & -c_{L_{0}}c_{M_{0}} & \left(N+H_{0}\right)c_{M_{0}}c_{L_{0}}\\ -s_{L_{0}}s_{M_{0}} & c_{L_{0}} & -c_{M_{0}}s_{L_{0}} & \left(N+H_{0}\right)c_{M_{0}}s_{L_{0}}\\ c_{M_{0}} & 0 & -s_{M_{0}} & \left(N\left(1-e^{2}\right)+H_{0}\right)s_{M_{0}}\\ 0 & 0 & 0 & 1 \end{array}\right]\;\times \;\\ \left[\begin{array}{cccc} c_{\varepsilon }c_{\beta } & -c_{\gamma }s_{\beta }+s_{\gamma }s_{\varepsilon }c_{\beta } & s_{\gamma }s_{\beta }+c_{\gamma }s_{\varepsilon }c_{\beta } & 0\\ c_{\varepsilon }s_{\beta } & c_{\gamma }c_{\beta }+s_{\gamma }s_{\varepsilon }s_{\beta } & -s_{\gamma }c_{\beta }+c_{\gamma }s_{\varepsilon }s_{\beta } & 0\\ -s_{\varepsilon } & s_{\gamma }c_{\varepsilon } & c_{\gamma }c_{\varepsilon } & 0\\ 0 & 0 & 0 & 1 \end{array}\right]\;\times \;\left[\begin{array}{cccc} c_{\Theta }s_{\Psi } & -s_{\Theta } & c_{\Theta }c_{\Psi } & 0\\ s_{\Psi }s_{\Theta } & c_{\Theta } & s_{\Theta }s_{\Psi } & 0\\ -c_{\psi } & 0 & s_{\Psi } & 0\\ 0 & 0 & 0 & 1 \end{array}\right]\;\times \;\left[\begin{array}{c} x_{c}\\ y_{c}\\ z_{c}\\ 1 \end{array}\right] \end{array}$$

where $c_{*}=\cos\left(*\right)$, $s_{*}=\sin\left(*\right)$.

Then we derive the geodetic coordinates of main target from earth centered earth fixed-world geodetic system (ECEF-WGS) transformation equations [19]:

(2) $$U=\arctan\frac{az_{e}}{b\sqrt{x_{e}^{2}+y_{e}^{2}}}$$

(3) $$L=\{\begin{array}{c} \arctan\frac{y_{e}}{x_{e}},\;when\;x_{e}>0\\ \frac{\pi }{2},\;when\;x_{e}=0,\;y_{e}>0\\ -\frac{\pi }{2},\;when\;x_{e}=0,\;y_{e}<0\\ \pi +\arctan\frac{y_{e}}{x_{e}},\;when\;x_{e}<0,\;y_{e}>0\\ -\pi +\arctan\frac{y_{e}}{x_{e}},\;when\;x_{e}<0,\;y_{e}<0 \end{array}$$

(4) $$M=\arctan\frac{z_{e}+be^{2}\sin^{3}U}{\sqrt{x_{e}^{2}+y_{e}^{2}}-ae^{2}\cos^{3}U}$$

(5) $$H=\frac{\sqrt{x_{e}^{2}+y_{e}^{2}}}{\cos M}-N$$

In Equations (1)–(5), the semi-major axis of ellipsoid is a=6378137.0m, the semi-minor axis of ellipsoid is b=6356752.0m, the first eccentricity of spheroid $e=\sqrt{a^{2}-b^{2}}/a$, the second eccentricity of spheroid is $e^{\prime }=\sqrt{a^{2}-b^{2}}/b$, and the radius of spheroid curvature in the prime vertical is $N=a/\sqrt{1-e^{2}sin^{2}M}$.

**Figure 8:**
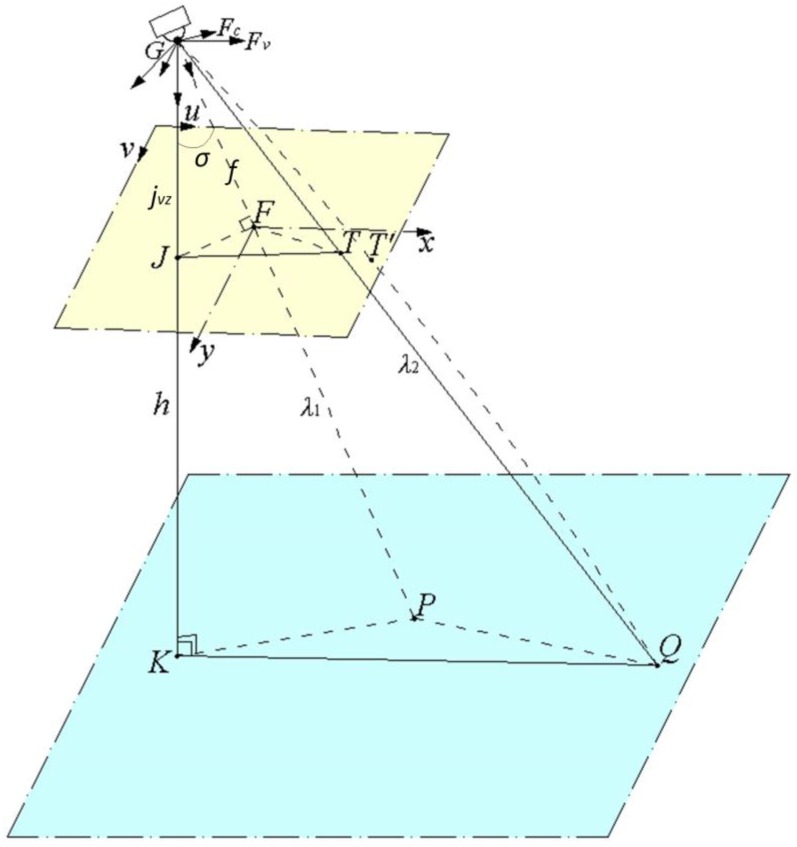
The location of any target in image model.

The key of sub-target geo-location is to build a geometrical geo-location model. The coordinates $\left(x_{c},y_{c},z_{c}\right)$ of sub-targets in the camera frame are solved on the basis of their pixel coordinates, and then their geodesic coordinates are calculated in accordance with the coordinate transformation Equations (1)–(5), Suppose the ground area corresponding to a single image is flat and the relative altitudes between targets and electro-optical stabilized imaging system are the same. Based on the image forming principles for single-plane array charge coupled device (CCD) sensors, a multi-target geo-location model can be established, as shown in Figure 8.

Suppose that no image distortion exists, the image point T of main target P is at the image center, and the three points, namely projection center G, sub-target Q and its image point T, are on the same line. Then a pin-hole imaging model will be formed and the altitude of a target relative to electro-optical stabilized imaging system will be:

(6) $$h=\lambda_{1}\cos\alpha =\lambda_{2}\cos\beta$$

where: h is the relative altitude, $\lambda_{1}$ is the distance from electro-optical stabilized imaging system to main target, and $\lambda_{2}$ is the distance from electro-optical stabilized imaging system to a sub-target.

Suppose the line-of-sight (LOS) vectors of the main target P, the sub-target Q and the point K beneath the camera are $\vec{s}=\vec{GF}$, $\vec{t}=\vec{GT}$, $\vec{j}=\vec{GJ}$ respectively, α is the angle between $\vec{s}$ and $\vec{j}$, and β is the angle between $\vec{t}$ and $\vec{j}$, then [20]:

(7) $$\cos\alpha =\frac{\vec{s}•\vec{j}}{\left\|\vec{s}\right\|\;\left\|\vec{j}\right\|}$$

(8) $$\cos\beta =\frac{\vec{t}•\vec{j}}{\left\|\vec{t}\right\|\;\left\|\vec{j}\right\|}$$

$F_{c}$ is the basis vectors for camera frame in a 3-dimensional vector space, the coordinates of LOS vectors $\vec{s}$ and $\vec{t}$ in the camera frame are given by Equations (9) and (10):

(9) $$\vec{s}=F_{c}^{T}\left[\begin{array}{c} 0\\ 0\\ f \end{array}\right]$$

(10) $$\vec{t}=F_{c}^{T}\left[\begin{array}{c} u-u_{0}\\ v-v_{0}\\ f \end{array}\right]$$

where f is the camera focal length, the unit is mm. The pixel coordinate of the point F is $\left(u_{0},v_{0}\right)$. The pixel coordinates of the point T is (u,v).

$F_{v}$ is the basis vectors for vehicle frame in a 3-dimensional vector space. In vehicle frame, the LOS vector $\vec{j}$ goes down axis $z_{v}$, the coordinates of $\vec{j}$ in vehicle frame is given by Equation (11):

(11) $$\vec{j}=F_{v}^{T}j_{v}=F_{v}^{T}\left[\begin{array}{c} 0\\ 0\\ j_{v_{z}} \end{array}\right]$$

The coordinates of $\vec{j}$ in the camera frame are solved as:

(12) $$j_{c}=R_{cv}j_{v}=R_{cb}R_{bv}j_{v}=\left[\begin{array}{ccc} c_{\Theta }c_{\Psi } & c_{\Psi }s_{\Theta } & -s_{\Psi }\\ -s_{\Theta } & c_{\Theta } & 0\\ c_{\Theta }s_{\Psi } & s_{\Theta }s_{\Psi } & c_{\Psi } \end{array}\right]\;\left[\begin{array}{ccc} c_{\varepsilon }c_{\beta } & c_{\varepsilon }s_{\beta } & -s_{\varepsilon }\\ c_{\beta }s_{\gamma }s_{\varepsilon }-c_{r}s_{\beta } & c_{r}c_{\beta }+s_{r}s_{\varepsilon }s_{\beta } & c_{\varepsilon }s_{\gamma }\\ s_{r}s_{\beta }+s_{r}s_{\beta }s_{\varepsilon } & c_{r}s_{\varepsilon }s_{\beta }-c_{\beta }s_{\gamma } & c_{r}c_{\varepsilon } \end{array}\right]\;j_{v}$$

where $c_{*}=\cos\left(*\right)$, $s_{*}=\sin\left(*\right)$.

$R_{bv}$ is the rotation matrix transformation from the vehicle frame to the body frame. $R_{cb}$ is the rotation matrix transformation from the body frame to the camera frame. $R_{cv}$ is the rotation matrix transformation from the vehicle frame to the camera frame.

σ is the angle between the $z_{v}$ axis of the vehicle frame and the $z_{c}$ axis of the camera frame. According to the geometric relationship in Figure 6, we obtain:

(13) $$\left\|\vec{j}\right\|=j_{v_{z}}$$

(14) $$f=j_{v_{z}}\cos\sigma$$

Using Euler parameters, or quaternions, we have the definition:

(15) $$\eta =\cos\frac{\sigma }{2}$$

It can also be shown that [21]:

(16) $$\eta =\pm \frac{1}{2}{\left(1+\mathrm{tr}C_{cv}\right)}^{\frac{1}{2}}$$

This may be manipulated into:

(17) $$2\eta^{2}-1=\frac{\mathrm{tr}C_{cv}-1}{2}$$

Therefore:

(18) $$j_{v_{z}}=\frac{f}{\cos\sigma }=\frac{f}{2\cos^{2}\left(\frac{\sigma }{2}\right)-1}=\frac{f}{2\eta^{2}-1}$$

and it follows that:

(19) $$j_{v_{z}}=\frac{2f}{trC_{cv}-1}$$

By substituting the $j_{v_{z}}$ value into Equations (11) and (12), the coordinate $j_{c}$ of $\vec{j}$ in the camera frame can be obtained. Then $j_{c}$ is substituted into Equations (7) and (8), to obtain cosα and cosβ. Finally, according to the known main target distance $\lambda_{1}$ and Equation (6), the relative altitude h and the sub-target distance $\lambda_{2}$ can be determined. Based on the sub-target distance $\lambda_{2}$ and the LOS vector $\vec{t}$ of the sub-target in the camera frame, the coordinates of the sub-target in this frame can be determined:

(20) $$\left[\begin{array}{c} x_{c}\\ y_{c}\\ z_{c} \end{array}\right]=\lambda_{2}\frac{\vec{t}}{\left\|\vec{t}\right\|}$$

Finally, the geodetic longitude L, the geodetic latitude M and geodetic height H of the sub-target can be calculated by substituting $x_{c}$, $y_{c}$ and $z_{c}$ into Equations (1)–(5).

### 3.3. Targets Tracking

The operator selects multiple targets in the first image and then these targets are tracked using the tracking algorithm. In recent years, many excellent tracking algorithms were proposed [22,23,24,25,26,27,28,29,30,31]. Due to the limited hardware resources in the TMS320DM642 (Texas Instruments Incorporated, Dallas, TX, USA), the tracking algorithm for UAV applications must be simple as well as highly efficient to meet the performance demands of real-time multiple target tracking. We use a simple two stage method to improve the real-time performance of the correlation tracking algorithm described in [13]. The main improvements are as follows:

In the low resolution stage, we calculate the average of four adjacent pixels in the original image to generate a low resolution image, whose resolution is half that of the original image. The low resolution template is generated in the same way. The formula of the normalized cross correlation (NCC) algorithm is as follows:

(21) $$R(u,v)=\frac{\sum_{x=1}^{m}\sum_{y=1}^{m}T\left(x,y\right)S\left(x+u,y+v\right)}{\sqrt{\sum_{x=1}^{m}\sum_{y=1}^{m}T^{2}\left(x,y\right)}\sqrt{\sum_{x=1}^{m}\sum_{y=1}^{m}S^{2}\left(x+u,y+v\right)}}$$

where the size of low resolution template T is m×m, the size of the low resolution search area S is n×n, (u,v) is the left corner point coordinate in the search area, 0≤u≤ n−m, 0≤v≤ n−m. T is moving on the S during the matching operation. When R(u,v) reaches the maximum value $R\left(u_{0},v_{0}\right)$, the point $\left(u_{0},v_{0}\right)$ is the best matching point in the low resolution search area.

In the original stage, we only need to search a small area in the original image. The size of the template is 2 m×2 m. The size of the small search area is (2 m+2)×(2 m+2). The left corner point coordinate in the search area is $\left(2u_{0}-1,2v_{0}-1\right)$. Then, the best matching point in the original image can be calculated using the NCC algorithm. In our implementation, m is set to 28, n is set to 46.

In [13], the time of template image matching is implemented is 0.62 ms. After improvement, it consumes 0.4 ms on the multi-target localization circuit board. The improved method can meet the real-time requirements of multi-target tracking.

## 4. Methods to Improve the Accuracy of Multi-Target Localization

### 4.1. Distortion Correction

The above multi-target geo-location model is established under the assumption that image distortion does not exist. In fact, due to the lens design and manufacturing errors of imaging systems, the image will be distorted [32,33,34,35], so the projection rays between the image point and object point can't completely meet the requirement of linear propagation in the total field of view (TFOV) and instead, will bend to some extent. As shown in Figure 6, the image point of the main target moves from the ideal position T to a distorted point position $T^{\prime }$, and the three points, namely projection center G, image point $T^{\prime }$ and object point Q, are not in a straight line. Thus it does not conform to the ideal pinhole imaging model. The calculation of target geo-location data based on the ideal pinhole imaging model will lead to a big error, so the lens distortion must be corrected. Distortion correction involves at first deriving the position T of the target in the ideal image from its image point $T^{\prime }$ in the distorted image according to the distortion model of camera, and then to calculate the geodetic coordinates of the target by using the pixel coordinates of the ideal image point T.

Real-time distortion correction of a zoom lens is impossible by using the calibration methods because the tedious calibration process has to be repeated again if the focal length is changed. In this research, we divide the zoom lens distortion procedures into two steps: lens distortion parameter estimation in the laboratory and real-time zoom lens distortion correction on the UAV.

#### 4.1.1. Lens Distortion Parameter Estimation in the Laboratory

Lens distortion parameter estimation is performed in the laboratory. We use UAV electro-optical stabilized imaging system to take images which contain a chessboard pattern in the laboratory. The lines in the chessboard pattern are straight in the real world, but the images generally contain curved lines caused by the lens distortion. We take lots of images with different focal lengths of a zoom lens. We use the images to construct the distortion parameter table, which is shown in Figure 3.

We extract the chessboard image edges using the Canny edge detector. The thresholds of the Canny edge detector are provided in terms of percentages of the gradient norm of the image.

For a zoom lens, the typical range of distortion coefficient $k_{1}$ is given by [$-\frac{1}{D^{2}},\;\frac{1}{D^{2}}$], D is the diagonal of the image [36]. In pixel coordinates, the image size is w×h pixels, the distortion center is $\left(u_{0},v_{0}\right)$, the image center is $\left(u_{c},v_{c}\right)$, where $u_{c}=0.5w$, $v_{c}=0.5h$. The range of $u_{0}$ is

$\left[u_{c}-0.05w,u_{c}+0.05w\right]$. The range of $v_{0}$ is $\left[v_{c}-0.05h,v_{c}+0.05h\right]$ [37,38].

We sampled $N_{2}$ samples of $u_{0}$ in the range of $\left[u_{c}-0.05w,\;u_{c}+0.05w\right]$. We sampled $N_{3}$ samples of $v_{0}$ in the range of $\left[v_{c}-0.05h,v_{c}+0.05h\right]$. We sample $N_{1}$ samples of $k_{1}$ in the range of [$-\frac{1}{D^{2}},\frac{1}{D^{2}}$] in each distortion center $\left(u_{0},v_{0}\right)$, so $N_{1}\times N_{2}\times N_{3}$ possible distortion parameters are generated from these samples in a certain camera focal length. The distortion parameter $\left(k_{1}^{i},u_{0}^{j},v_{0}^{p}\right)$ is shown in Equation (22) to Equation (24):

(22) $$k_{1}^{i}=-\frac{1}{D^{2}}+i\times \delta k_{1}$$

(23) $$u_{0}^{j}=0.45w+j\times \delta u_{0}$$

(24) $$v_{0}^{p}=0.45h+p\times \delta v_{0}$$

where $i=1,2,\ldots ,N_{1}$; $j=1,2,\ldots ,N_{2}$; $p=1,2,\ldots ,N_{3}$; $\delta k_{1}=\frac{2}{N_{1}D^{2}}$; $\delta u_{0}=\frac{0.1w}{N_{2}}$; $\delta v_{0}=\frac{0.1h}{N_{3}}$;

For each distortion parameter $\left(k_{1}^{i},u_{0}^{j},v_{0}^{p}\right)$, the pixel coordinates of the corrected chessboard image’s edge points $\left(u_{n},v_{n}\right)$ are computed by using Equation (25) to Equation (28):

(25) $$x_{d}=\left(u_{d}-u_{0}\right)d_{x}$$

(26) $$y_{d}=\left(v_{d}-v_{0}\right)d_{y}$$

(27) $$u_{n}=u_{0}+\frac{x_{d}}{d_{x}\left(1+k_{1}x_{d}^{2}+k_{1}y_{d}^{2}\right)}$$

(28) $$v_{n}=v_{0}+\frac{y_{d}}{d_{y}\left(1+k_{1}x_{d}^{2}+k_{1}y_{d}^{2}\right)}$$

$d_{x}$, $d_{y}$ are pixel size, which units are µm. The distortion center is $\left(u_{0},v_{0}\right)$, and the unit is pixels. The pixel coordinates of the distorted image are $\left(u_{d},v_{d}\right)$, in units of pixels. The pixel coordinates of the undistorted (corrected) image is $\left(u_{n},v_{n}\right)$, and the units are pixels. $\left(x_{d},y_{d}\right)$ is the projection coordinates of a distorted point, which units are μm.

The gradient direction $\alpha \left(u_{n},v_{n}\right)$ of the corrected chessboard image’s edge points $\left(u_{n},v_{n}\right)$ are computed using Equation (29) to Equation (31):

(29) $$G_{u}=\frac{I_{v_{n},u_{n}+1}-I_{v_{n},u_{n}}+I_{v_{n}+1,u_{n}+1}-I_{v_{n}+1,u_{n}}}{2}$$

(30) $$G_{v}=\frac{I_{v_{n}+1,u_{n}}-I_{v_{n},u_{n}}+I_{v_{n}+1,u_{n}+1}-I_{v_{n},u_{n}+1}}{2}$$

(31) $$\alpha \left(u_{n},v_{n}\right)=\arctan\left(\frac{G_{v}}{G_{u}}\right)$$

I is the chessboard image brightness value, $G_{u}$, $G_{v}$ are the first-order derivatives of the corrected image’s edge points brightness.

We compute the Hough transform of the corrected chessboard image. The N strongest peaks in the Hough transform correspond to the most distinct lines. The distance between a line $N_{q}$ and the origin is dist(q). The orientation of a line $N_{q}$ is β(q), where q=1,2,..., N.

If the angular difference between the edge point orientation $\alpha \left(u_{n},v_{n}\right)$ and the line $N_{q}$ orientation β(q) is less than a certain threshold (in our implementation, it is set to 2°. This threshold can meet the distortion correction accuracy requirements. We compute the distance $d_{q}$ from edge point $\left(u_{n},v_{n}\right)$ to the line $N_{q}$:

(32) $$d_{q}=\left|u_{n}\cos\left(\beta \left(q\right)\right)+v_{n}\sin\left(\beta \left(q\right)\right)-dist\left(q\right)\right|$$

If $d_{q}$ is less than a certain threshold. In our implementation, it is set to 2 pixels. This threshold can meet the distortion correction accuracy requirements. The edge point $\left(u_{n},v_{n}\right)$ votes for the line $N_{q}$, the votes of the edge point $\left(u_{n},v_{n}\right)$ is:

(33) $$\mathrm{votes}=\frac{1}{1+d_{q}}$$

We compute the sum of all edge points votes. In this focal length, the best distortion parameters $\left(k_{1},u_{0},v_{0}\right)$ are obtained by maximizing the straightness measure function:

(34) $$max\left\{\overset{N}{\underset{q=1}{\sum }}votes\left(dist\left(q\right),\beta \left(q\right),k_{1}^{i},u_{0}^{j},v_{0}^{p}\right)\right\}$$

where $\mathrm{votes}\left(dist\left(q\right),\beta \left(q\right),k_{1}^{i},u_{0}^{i},v_{0}^{p}\right)$ is the votes of the line $N_{q}$ in the corrected chessboard image using distortion parameter $\left(k_{1}^{i},u_{0}^{j},v_{0}^{p}\right)$.

We apply the above algorithm to calibrate the best distortion parameter $\left(k_{1},u_{0},v_{0}\right)$ with different lens focal lengths. Then, the best zoom lens distortion parameters $\left(k_{1},u_{0},v_{0}\right)$ in all focal lengths are gained through curve fitting using Matlab Tools. We store the distortion parameter talbe for all focal length in the flash chip on the multi-target geo-location and tracking circuit board.

#### 4.1.2. Real-Time Lens Distortion Correction on the UAV

The zoom lens is connected to the potentiometer through the gears. The relationship between focal length and resistance has been calibrated in the laboratory. We can get the focal length by measuring the resistance value of the potentiometer in real-time. During the flight of the UAV, we use the focal length measuring sensor (model: S10HP-3 3-turn Potentiometer, SAKAE, Nagoya, Japan, resistance error: ±1%) to measure the camera focal length and we find the distortion parameter $\left(k_{1}^{i},u_{0}^{j},v_{0}^{p}\right)$ in the flash chip on the multi-target localization circuit board. The pixel coordinates $\left(u_{n},v_{n}\right)$ of the corrected real-time image are computed using Equation (25) to Equation (28). We use $\left(u_{n},v_{n}\right)$ to calculate the geodetic coordinates of the targets.

### 4.2. RLS Filter

For stationary targets on the ground, the location result in different frames should be the same. Therefore, a popular technique to remove the estimation error is to use a recursive least squares (RLS) filter. The RLS filter minimizes the average squared error of the estimate. The RLS filter uses an algorithm that only requires a scalar division at each step, making the RLS filter suitable for real-time implementation, so we use RLS to reduce the standard deviation and improve the accuracy of multiple stationary target localization.

**Figure 9:**
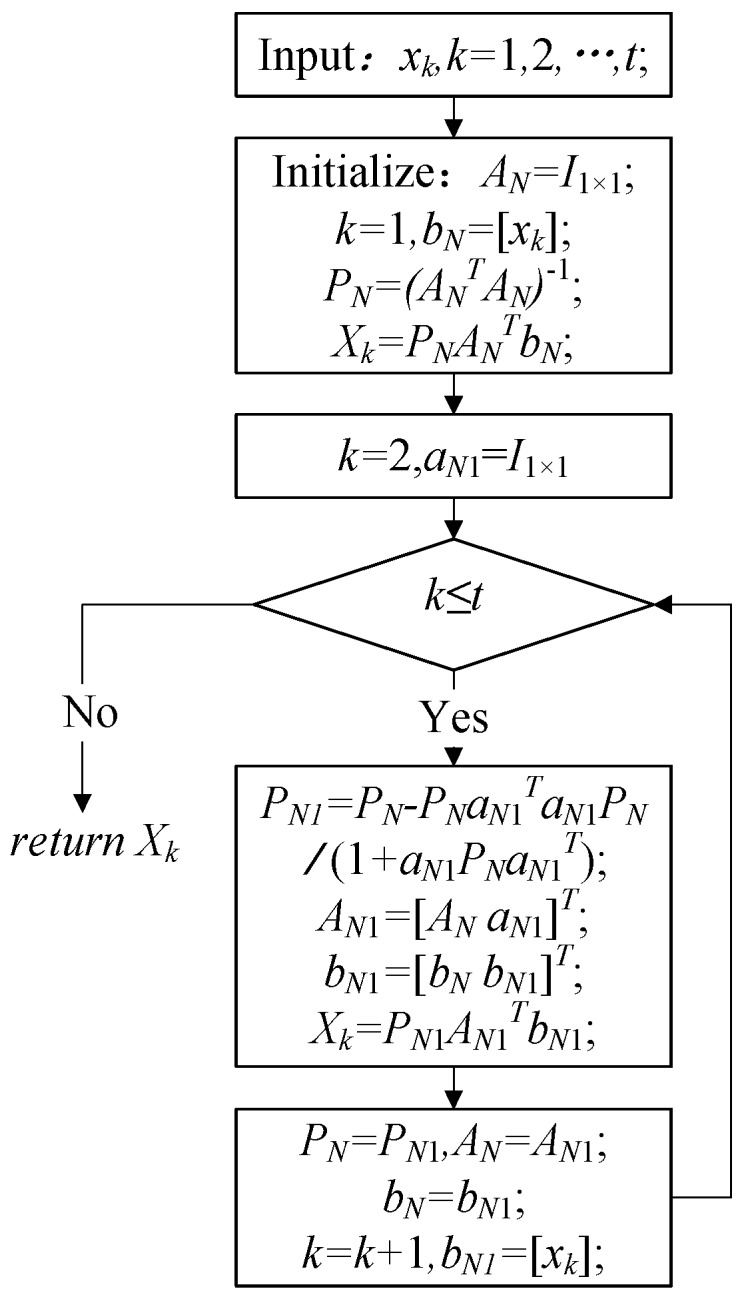
Flowchart of the RLS algorithm.

Suppose the original geo-location data of t images are $x_{k}$ (k=1,2,…,t). The RLS algorithm flowchart is shown in Figure 9. In Figure 9, $I_{1X1}$ is a 1×1 unit matrix. After the RLS filtration of the original data $x_{k}$, the obtained data are $X_{k}$ (k=1, 2,… t), where $X_{k}$ can be longitude L, latitude M or geodetic height H.

The GPS data (coordinates of the UAV) refresh rate is 1 Hz, but the video frame rate is usually above 25 Hz. To raise the convergence rate of the RLS algorithm, when the UAV speed is known, the coordinates of the UAV at the corresponding time can be determined through dead reckoning. In the WGS-84 ECEF, the coordinates of the UAV are [39]:

(35) $$x_{e}=x_{e0}+\int_{0}^{n}V_{x}dt$$

(36) $$y_{e}=y_{e0}+\int_{0}^{n}V_{y}dt$$

where $\left(x_{e0},y_{e0}\right)$ are the coordinates at the initial time, and $V_{x}$ is UAV speed in direction X, $V_{y}$ is UAV speed in direction Y. The influence of the UAV geodetic coordinate position and speed on the reckoned coordinates of the UAV is analyzed as follows:

(1) In the WGS-84 geodetic frame, the higher the latitude of the UAV is, the smaller the projection of 1° longitude onto the horizontal direction. Therefore, in the high latitude area, the measurement accuracy of GPS is high, and the accuracy of the reckoned coordinates is high.
(2) The smaller the UAV speed is, the smaller the distance UAV moves in the same time interval, the higher accuracy of the reckoned coordinates is.

According to Equations (35) and (36), the error resulting from the updating rate of GPS data can be compensated to converge the RLS algorithm rapidly to a stable value. Therefore, we can geo-locate multiple stationary ground targets quickly and accurately.

## 5. Experiments and Discussion

The targets location data were obtained during a UAV flight in real time. The evaluation is based on UAV videos captured from a Changji highway from 9:40 to 11:10. The resolution of the videos is 1024 × 768 and the frame rate is 25 frames per second (fps).

### 5.1. The Zoom Lens Distortion Parameter Estimation Results

**Figure 10:**
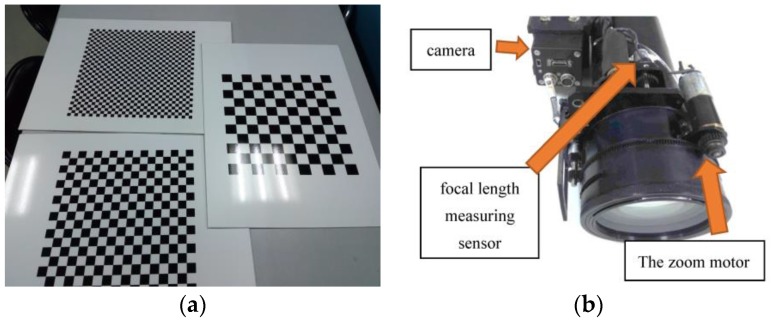
(**a**) A plane containing a chessboard pattern; (**b**) The zoom lens camera (not yet mount in the electro-optical stabilized imaging system).

To evaluate the proposed lens distortion parameters estimation approach, we use a plane containing a chessboard pattern and a zoom lens camera which are shown in Figure 10. The size of the pattern is 450 mm×450 mm. We take the images which contained the chessboard pattern for several focal lengths: f=5, 8, 10, 15, 20, 25, 30, 40, 50, 60, 70, 80, 90, 100. Then, we perform the lens distortion parameters estimation approach in Section 4.1 to estimate the lens distortion parameters.

**Figure 11:**
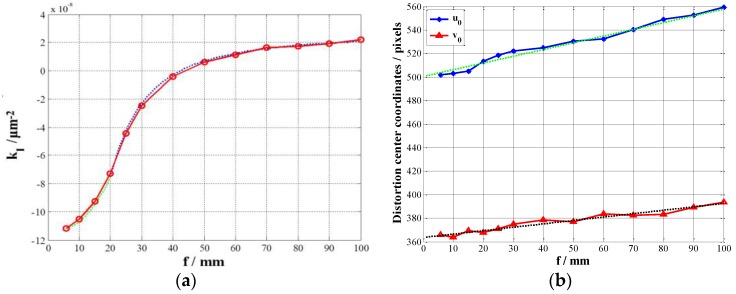
(**a**) The relationships between distortion coefficient $k_{1}$ and the focal length f; (**b**) The relationships between the distortion center $\left(u_{0},v_{0}\right)$ and the focal length f.

The relationships between the distortion coefficient $k_{1}$ and the focal length f are shown in Figure 11a. The relationships between the distortion center $\left(u_{0},v_{0}\right)$ and the focal length f are shown in Figure 11b.

We fit the curve between the data. If 5.8 mm≤f≤20 mm The relationships between the distortion coefficient $k_{1}$ and the focal length f is shown in Equation (37):

(37) $$k_{1}\left(f\right)=\rho \times f^{2}+\sigma \times f+\tau$$

where $\rho =1.369\times 10^{-10}$, $\sigma =-1\times 10^{-9}$, $\tau =-1.108\times 10^{-7}$.

If 20 mm≤f≤100 mm. The relationships between the distortion coefficient $k_{1}$ and the focal length f is shown in Equation (38):

(38) $$k_{1}\left(f\right)=\frac{\delta }{\left(f+\eta \right)}+\psi$$

where $\delta =-5.245\times 10^{-5}$, η=2.768, $\psi =2.564\times 10^{-8}$.

We fit the curve between the data. The relationships between the distortion center $\left(u_{0},v_{0}\right)$ and the focal length f is shown in Equations (39) and (40):

(39) $$u_{0}\left(f\right)=\lambda_{1}\times f+\mu_{1}$$

(40) $$v_{0}\left(f\right)=\lambda_{2}\times f+\mu_{2}$$

where $\lambda_{1}=0.5722$, $\mu_{1}=500.4904$, $\lambda_{2}=0.2897$, $\mu_{2}=363.5341$.

Based on the above fitting formula, we calculate the zoom lens distortion parameter $\left(k_{1}^{i},u_{0}^{j},v_{0}^{p}\right)$ in all focal length to construct the distortion parameter table in the laboratory. We store the distortion parameter table in the flash chip in multi-target localization circuit board.

### 5.2. Targets Location Experimental Design and Instrument Description

This test is divided into the following four parts:

(1) Monte Carlo simulation analysis of multi-target geo-location error. Through this analysis, the expected error of multi-target geo-location can be determined.
(2) Geo-location test of a single aerial image. We substitute an actual aerial image and its position/attitude data into multi-target geo-location program for target location resolution. We use a high-precision GPS receiver to measure the geo-location data of various ground targets as the nominal values for target geo-location. We compare the calculated values with these nominal values to obtain the multi-target geo-location accuracy of the image. We correct the geo-location error arising from lens distortion, and then compare the calculated geodesic coordinates of each target with its nominal geodesic coordinates to determine the multi-target geo-location accuracy after the distortion correction.
(3) Geo-location test of multi-frame aerial images. For many stationary ground targets, we use the RLS algorithm to adaptively estimate the multi-frame image geo-location data and then by comparing the RLS filtration results with nominal values, we determine the target geo-location accuracy after the RLS filtration.
(4) Real-time geo-location and tracking of multiple ground-based moving targets. We derive the motion trail of each target from the geo-location data and time interval of every image. Then we calculate the speed of each target.

Here, a GPS receiver of the Geo Explorer 3000 series is used for ground measurements. This instrument has 14 channels, including 12 L1 codes and carriers and two satellite-based augmentation systems (SBAS). It is integrated with real-time two-channel SBAS tracking technology, and supports real-time differential correction. It can achieve real-time sub-meter geo-location accuracy—An accuracy of 50 cm is available through Trimble Delta Phase postprocessing.

### 5.3. Test 1: Monte Carlo Analysis of Multi-Target Geo-Location Accuracy Error

Error analysis is an important step to judge if a geo-location method is good or not. It is very difficult to analyze the target geo-location error through complete differential based on the measurement equation of airborne electro-optical stabilized imaging system, so Monte Carlo analysis is introduced to analyze the multi-target geo-location error. The Monte Carlo method is based on the law of great number and the Bernoulli’s theory [40]. On the basis of this method, a model of multi-target geo-location error can be established:

(41) $${\left[\Delta L\;\Delta M\;\Delta H\right]}^{T}=F^{\prime }(X)-F^{\prime }(X-\Delta X)$$

where: ΔL, ΔM and ΔH are the geo-location errors of each target, and ΔX is geo-location parameter error.

We use five aerial images (32 targets) for multi-target geo-location and distortion correction test. (eight targets in the 1st image, six targets in the 2nd image, five targets in the 3rd image, five targets in the 4th image, and eight targets in the 5th image). We use eight targets in the 1st image in Test 1. The image size is 1024 pixels × 768 pixels, and the pixel size is 5.5 μm × 5.5 μm. The position/attitude data of electro-optical stabilized imaging system collected through GPS, attitude measurement and laser finding at the time of image shoot are shown in Table 1. The root-mean-square error (RMSE) of each parameter is determined in accordance with the maximum nominal error stipulated in the relevant measurement equipment specifications.

Based on the nominal values and errors of above parameters, the sample model for 10,000 random variable arrays can be established in the Matlab software. By using the Equations (1)–(20) and (41), the geodetic coordinate RMSE of every target in this test can be determined through the Monte Carlo method, as shown in Table 2.

### 5.4. Test 2: Multi-Target Geo-Location Using a Single Aerial Image with Distortion Correction

The data in Table 1 are substituted into Equations (1)–(20) to calculate the geodesic coordinates of each target in a single image, as shown in Table 3.

The calculated geodetic coordinates of each target in Table 3 are compared with its nominal geodetic coordinates measured by GPS receiver on the ground, in order to obtain its geo-location error in a single image, as shown in Table 4. It is found that the geo-location error of each target is within the expected error range through the comparison between the data in Table 4 and those in Table 2. The geodetic height geo-location errors of all the targets are about 18 m—that’s basically in line with the assumption in the Section 3.2 that “the ground area corresponding to a single image is flat and the relative altitudes between targets and electro-optical stabilized imaging system are the same”.

The latitude and longitude geo-location errors of sub-targets are bigger than those of main targets for the following reasons: (1) the error arising from the slope distance difference between a main target and a sub-target. The longer the slope distance of a target to the image border, the bigger the geo-location error [39]; (2) the coordinate transformation error caused by the attitude measurement error and the angle measurement error (measured by the electro-optical stabilized imaging system) during the calculation of altitude and distance of a sub-target relative to the electro-optical stabilized imaging system; (3) the pixel coordinate error of a sub-target found in target detection; and (4) the pixel coordinate error of a sub-target caused by image distortion.

The geo-location errors of a target can be reduced in three ways: by reduction in the flight height $H_{0}$ or an increase in the platform elevation angle Ψ (the horizontal forward direction is 0°) to shorten the slope distance, which needs to consider the flight conditions; the selection of a high-precision attitude measuring system and the improvement in angle measurement accuracy of the electro-optical stabilized imaging system, for which one needs to consider the hardware cost; and the distortion correction. Therefore, the influence of distortion correction on multi-target geo-location accuracy will be mainly discussed.

Sub-target pixel coordinate error is mainly caused by image distortion. The correction method is discussed in Section 4.1 and Section 5.1. We calculate the corrected pixel coordinates using Equation (25) to Equation (28). Then, we use corrected pixel coordinates to calculate geodetic coordinates of target. Geo-location errors of multi-target after distortion correction are shown in Table 5.

It can be seen through the comparison between the data in Table 5 and those in Table 4 that, after the distortion correction, the latitude and longitude errors of each target are generally smaller than those before the correction, while the geo-location error of geodetic height remains basically unchanged. For the sub-targets farther from the image center, a more significant reduction in longitude and latitude errors can be obtained. Therefore, lens distortion correction can improve the geo-location accuracy of sub-targets and thus raise the overall accuracy of multi-target geo-location.

Target geo-location accuracy and missile hit accuracy are usually evaluated through the circular error probability (CEP) [41]. CEP is defined as the radius of a circle with the target point as its center and with the hit probability of 50%. In ECEF frame, the geo-location error along X direction is x. The geo-location error along Y direction is y. x and y can be calculated from longitude error and latitude error listed in Table 4 and Table 5. Suppose both x and y are subject to the normal distribution, the joint probability density function of (x,y) can be expressed as:

(42) $$f(x,y)=\frac{1}{2\pi \sigma_{x}\sigma_{y}\sqrt{1-\rho^{2}}}\times \exp\left\{-\frac{1}{2(1-\rho^{2})}\left[\frac{{\left(x-\mu_{x}\right)}^{2}}{\sigma_{x}^{2}}-\frac{2\rho \times (x-\mu_{x})\times (y-\mu_{y})}{\sigma_{x}\sigma_{y}}+\frac{{\left(y-\mu_{y}\right)}^{2}}{\sigma_{y}^{2}}\right]\right\}$$

where $\mu_{x}$ and $\mu_{y}$ are the mean geo-location errors along X and Y directions, respectively; $\sigma_{x}$ and $\sigma_{y}$ are the standard deviations of the geo-location errors along X and Y directions, respectively; ρ is the correlation coefficient of geo-location errors along X and Y directions, 0≤| ρ|≤1.

Suppose x=rcosθ, y=rsinθ and $r=\sqrt{x^{2}+y^{2}}$, the R satisfying the following equation will be CEP:

(43) $$\frac{1}{2\pi \sigma_{x}\sigma_{y}\sqrt{1-\rho^{2}}}\int_{0}^{R}\int_{0}^{2\pi }r\exp\left\{-\frac{1}{2(1-\rho^{2})}\left[\frac{{\left(rc_{\theta }-\mu_{x}\right)}^{2}}{\sigma_{x}^{2}}-\frac{2\rho (rc_{\theta }-\mu_{x})(rs_{\theta }-\mu_{y})}{\sigma_{x}\sigma_{y}}+\frac{{\left(rs_{\theta }-\mu_{y}\right)}^{2}}{\sigma_{y}^{2}}\right]\right\}drd\theta =0.5$$

where: $c_{\theta }=\cos\theta$, $s_{\theta }=\sin\theta$.

If the mean geo-location errors $\mu_{x}$, $\mu_{y}$ are unknown, they can be substituted by the sample mean geo-location errors ${\hat{\mu }}_{x}$, ${\hat{\mu }}_{y}$ respectively. If the standard deviations of the geo-location errors $\sigma_{x}$, $\sigma_{y}$ are unknown, they can be substituted by the sample standard deviation of the geo-location errors ${\hat{\sigma }}_{x}$, ${\hat{\sigma }}_{y}$ respectively. If the correlation coefficients of the geo-location errors ρ are unknown, they can be substituted by the sample correlation coefficients of the geo-location errors $\hat{\rho }$. If the total mean geo-location error $\mu_{r}$ is unknown, it can be substituted by the total sample mean geo-location error ${\hat{\mu }}_{r}$. If the total standard deviation of the geo-location error $\sigma_{r}$ is unknown, it can be substituted by the total sample standard deviation of the geo-location errors ${\hat{\sigma }}_{r}$.

Suppose the number of geo-location error samples is n: $\left(x_{1},y_{1}\right)$, $\left(x_{2},y_{2}\right)$, $\left(x_{3},y_{3}\right)$, … ,$\left(x_{n},y_{n}\right)$. The sample mean geo-location errors along X and Y directions are:

(44) $${\hat{\mu }}_{x}=\frac{1}{n}\sum_{i=1}^{n}x_{i}$$

(45) $${\hat{\mu }}_{y}=\frac{1}{n}\sum_{i=1}^{n}y_{i}$$

The total sample mean geo-location error is:

(46) $${\hat{\mu }}_{r}=\frac{1}{n}\sum_{i=1}^{n}r_{i}$$

The sample standard deviations of the geo-location errors along X and Y directions are:

(47) $${\hat{\sigma }}_{x}=\sqrt{\frac{1}{n-1}\sum_{i=1}^{n}{\left(x_{i}-{\hat{\mu }}_{x}\right)}^{2}}$$

(48) $${\hat{\sigma }}_{y}=\sqrt{\frac{1}{n-1}\sum_{i=1}^{n}{\left(y_{i}-{\hat{\mu }}_{y}\right)}^{2}}$$

The total sample standard deviations of the geo-location errors is:

(49) $${\hat{\sigma }}_{r}=\sqrt{\frac{1}{n-1}\sum_{i=1}^{n}{\left(r_{i}-{\hat{\mu }}_{r}\right)}^{2}}$$

The sample correlation coefficient of the geo-location errors is:

(50) $$\hat{\rho }=\frac{\sum_{i=1}^{n}\left[(x_{i}-{\hat{\mu }}_{x})\times (y_{i}-{\hat{\mu }}_{y})\right]}{\sqrt{\sum_{i=1}^{n}(x_{i}-{\hat{\mu }}_{x})\times \sum_{i=1}^{n}\left(y_{i}-{\hat{\mu }}_{y}\right)}}$$

When the number of geo-location error samples is more than 30, the confidence of CEP calculation result can reach 90% [41]. Therefore, through the multi-target geo-location and distortion correction test for five aerial images, 32 samples of target geo-location errors are obtained before and after the distortion correction, respectively, including eight in the 1st image, six in the 2nd image, five in the 3rd image, five in the 4th image, and eight in the 5th image. The normality test and independence test of sample data reveal that, the samples conform to normal distribution but are not independent (the sample correlation coefficient before the distortion correction is ρ^1=0.6618, and the sample correlation coefficient after the distortion correction is ρ^2=0.5607).

Geo-location errors of the eight targets in the 1st image (which are parts of 32 targets) before the correction are shown in Table 4. Geo-location errors of the eight targets in the 1st image (which are parts of 32 targets) after the correction are shown in Table 5.

**Figure 12:**
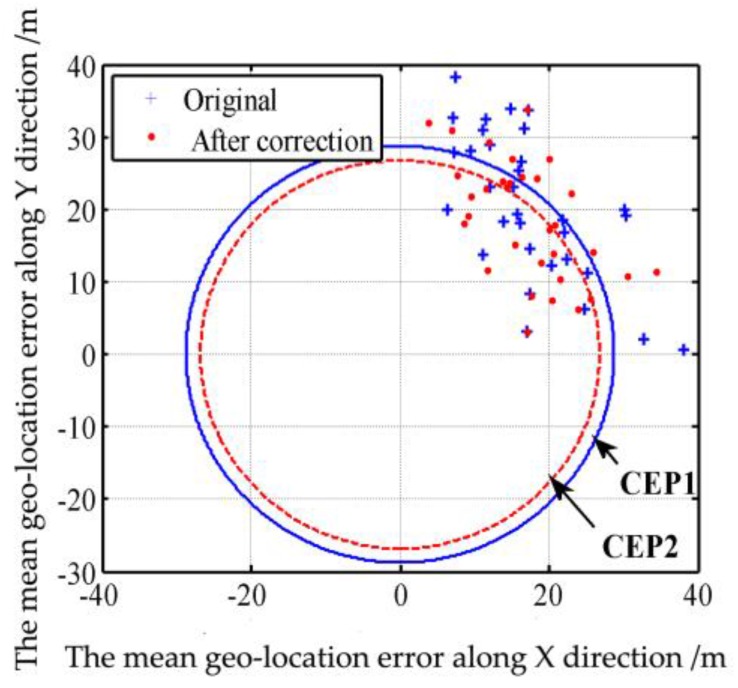
Sample distribution of target location error before and after distortion correction.

The multi-target geo-location errors before the distortion correction are processed through the Equations (43)–(50) to calculate the CEP, whereas the mean geo-location errors along the X and Y directions are 17.41 m and 20.34 m, respectively, the standard deviations of the geo-location errors along the X and Y directions are 7.77 m and 10.05 m, respectively, and the total mean geo-location error and total standard deviation are 28.98 m and 6.08 m, respectively. The calculation of Equation (46) through numerical integration finds that, among the 32 samples, 16 are in a circle with a radius of 28.74 m, which means the probability is 50%. The sizes of data samples inside and outside the solid circle in Figure 10 also show that, the CEP1 of multi-target geo-location before the distortion correction is 28.74 m. Then the multi-target geo-location errors after the distortion correction are processed through Equations (43)–(50) to calculate the CEP, where the mean geo-location errors along X and Y directions are 17.04 m and 18.63 m, respectively, the standard deviations of the geo-location errors along X and Y directions are 6.86 m and 8.25 m, respectively, and the total mean geo-location error and total standard deviation are 26.91 m and 5.31 m, respectively. The calculation of Equation (43) through numerical integration finds that, among the 32 samples, 16 are in a circle with a radius of 26.80 m, which means the probability is 50%. The sizes of samples inside and outside the dotted circle in Figure 10 also show that, the CEP2 of multi-target geo-location after the distortion correction is 26.80 m, 7% smaller than that before the distortion correction. Note: We use original geo-location error (32 samples scattered in the four quadrants) to calculate the CEP circle. Because there are 32 samples scattered in the four quadrants. It’s too scattered and not conducive to the analysis of the error. Therefore, we take the absolute value of each geo-location error, so all points are in the first quadrant in Figure 12.

In order to compare the performance of our multi-target geo-location algorithm with that of other algorithms, these localization accuracy results have been compared with the accuracies of geo-location methods reported in [7,8,9], as shown in the Table 6. It can be seen from the Table 6 that, the target geo-location accuracy in this paper is close to that reported in [7]. However, the geo-location accuracy in [7] depends on the distance between the projection centers of two consecutive images (namely baseline length). To obtain higher geo-location accuracy, the baseline length shall be longer, so in [7], the time interval between two consecutive images used for geo-location is quite big. Meanwhile, as the SIFT algorithm is needed to extract feature points from multi-frame images for the purpose of 3D reconstruction, the algorithm in [7] has a heavy calculation load in prejudice of real-time implementation. In [8], the geo-location accuracy is about 20 m before filtering. The real-time geo-location accuracy of the two methods is almost the same. However, our UAV flight altitude is much higher than that in [8]. In [9], geo-location accuracy is about 39.1 m before compensation. Our real-time geo-location accuracy is higher than in [9]. In [9], after the UAV flies many around in an orbit, the geo-location accuracy can be increased to 8.58 m. However, this accuracy cannot be obtained in real-time. The algorithms in [7,8,9] are implemented on a computer in the ground station. Since the images are transmitted to, and processed on a computer in the ground station, a delay occurs between the data capture moment and the time of completion of processing. In comparison, the geo-location algorithm in this paper is programmed and implemented on multi-target localization circuit board (model: THX-IMAGE-PROC-02, Changchun Institute of Optics, Fine Mechanics and Physics, Chinese Academy of Sciences, Changchun, China) with a TMS320DM642@ 720-M Hz Clock Rate and 32-Bit Instructions/Cycle and 1 GB DDR. It consumes 0.4 ms on average when calculating the geo-location of a single target and, at the same time, correcting zoom lens distortion. Therefore, this algorithm has great advantages in both geo-location accuracy and real-time performance. The multi-target location method in this paper can be widely applied in many areas such as UAVs and robots.

### 5.5. Test 3: RLS Filter for Geo-Location Data of Multiple Stationary Ground Targets

**Figure 13:**
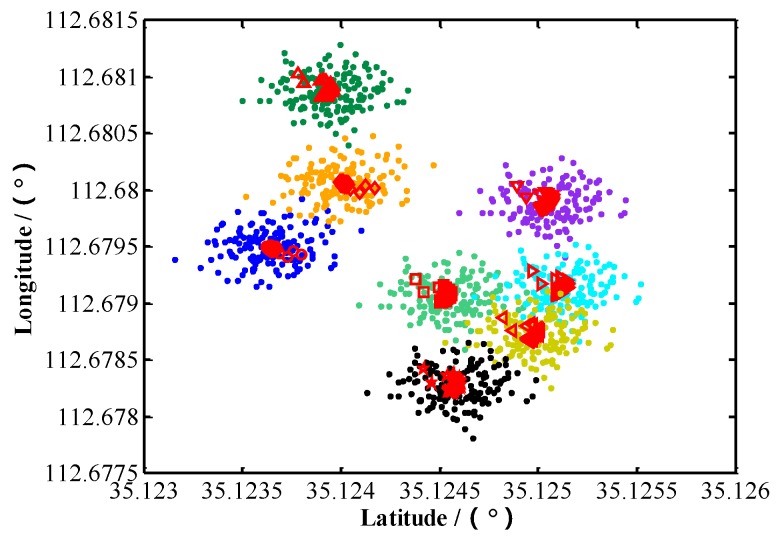
Localization results before and after RLS filtering.

**Figure 14:**
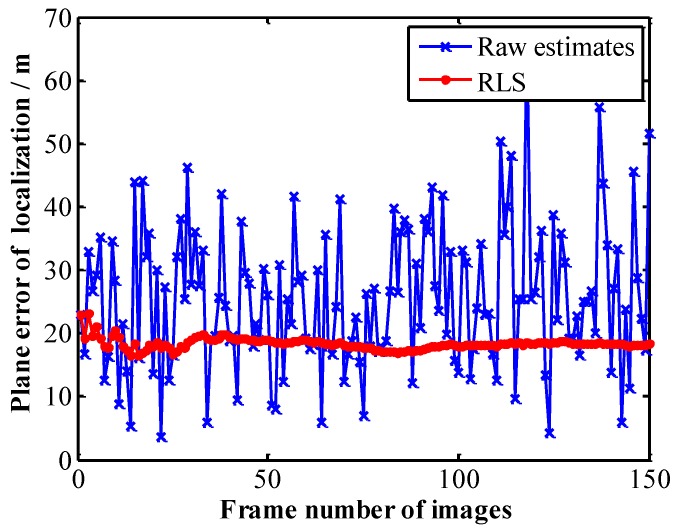
Plane localization errors after RLS filtering.

The targets in the above tests are all stationary ground targets. After lens distortion correction, we use the 1st aerial images (eight targets) as the initial frame for target tracking. For 150 frames starting from the 1st image, eight targets are tracked, respectively. The coordinates of the UAV are calculated using Equations (25) and (26). The update rate of UAV coordinate is synchronized with the camera frame rate. The geo-location data of each target are adaptively estimated by RLS algorithm respectively. The geo-location results of eight targets before and after RLS filtration are shown in Figure 13 (the dots “•” in different colors in Figure 13 represent original geo-location data of the eight targets, while □, ◁, ▷, ☆, ▽, ○, ◇ and △ represent the geo-location data of main target and sub-targets 1, 2, …, 7 after RLS filtration). It can be observed from Figure 13 that, after RLS filtration, the dispersion of geo-location data decreases sharply for each target, converging rapidly to a small area adjacent to the true position of the target. Figure 14 shows how the plane geo-location error of sub-target 2 changes with the number of image frames (the corresponding time) in the RLS filtration process. It can be seen from Figure 14 that after filtering the geo-location data of 100~150 images (the corresponding time is 3~5 s), the geo-location errors of the target have converged to a stable value. So, we can obtain a more accurate stationary target location immediately after 150 images (it is no longer necessary to run RLS).

By comparing the results after the stabilization of RLS filtration with the nominal geodetic coordinates of each target, the geo-location errors of geodetic coordinates of the targets after RLS filtration can be determined, as shown in Table 7.

It can be seen from the comparison of results between the data in Table 7 and those in Table 5 that, after RLS filtration, the longitude and latitude errors of each target are much smaller than the multi-target geo-location errors of a single image which is only processed by lens distortion correction. The geo-location error of geodetic height also decreases slightly.

After lens distortion correction, we use the other four aerial images (24 targets) as the initial frame for targets tracking, respectively (eight targets in the 1st image, six targets in the 2nd image, five targets in the 3rd image, five targets in the 4th image, and eight targets in the 5th image).

For 150 frames starting from the 2nd image, six targets are tracked, respectively. The geo-location data of each target are adaptively estimated by the RLS algorithm, respectively. For 150 frames starting from the 3rd image, five targets are tracked, respectively. The geo-location data of each target are adaptively estimated by the RLS algorithm, respectively. For 150 frames starting from the 4th image, five targets are tracked, respectively. The geo-location data of each target are adaptively estimated by the RLS algorithm, respectively. For 150 frames starting from the 5th image, eight targets are tracked, respectively. The geo-location data of each target are adaptively estimated by the RLS algorithm, respectively.

**Figure 15:**
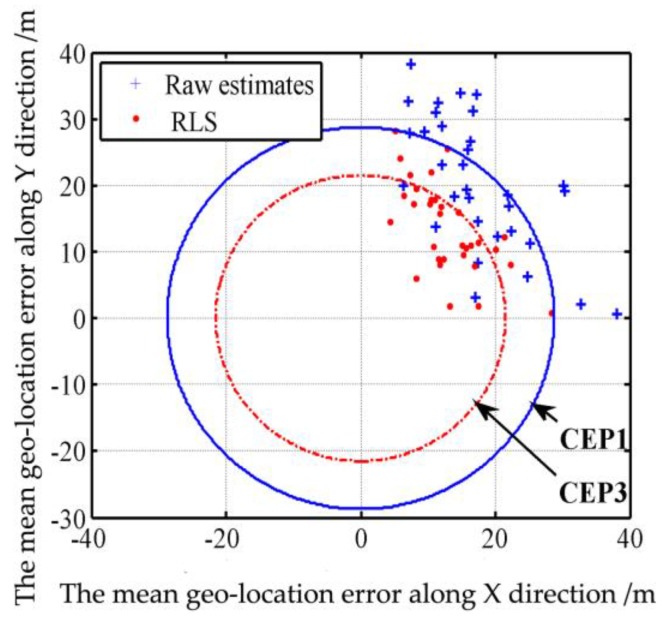
Sample distribution of target location before and after RLS.

The multi-target geo-location data after RLS filtration are processed through the Equations (43)–(50) to calculate the CEP, where the mean geo-location errors along the X and Y directions are 13.78 m and 14.53 m, respectively, and the standard deviations of the geo-location errors along the X and Y directions are 5.79 m and 7.34 m, respectively. Among the 32 samples obtained through numerical integration of Equation (43), 17 are in a circle with a radius of 21.52 m, which means the probability is 53%. The sizes of samples inside and outside the dotted circle in Figure 13 also show that, the CEP3 of multi-target geo-location after RLS filtration is 21.52 m, 25% smaller than the CEP1 of multi-target geo-location of a single image. Note: We use original geo-location error (32 samples scattered in the four quadrants) to calculate the CEP circle. Because there are 32 samples scattered in the four quadrants. It’s too scattered and not conducive to the analysis of the error. Therefore, we take the absolute value of each geo-location error, so all points are in the first quadrant in Figure 15.

### 5.6. Test 4: Real-Time Geo-Location and Tracking of Multiple Moving Ground Targets

**Figure 16:**
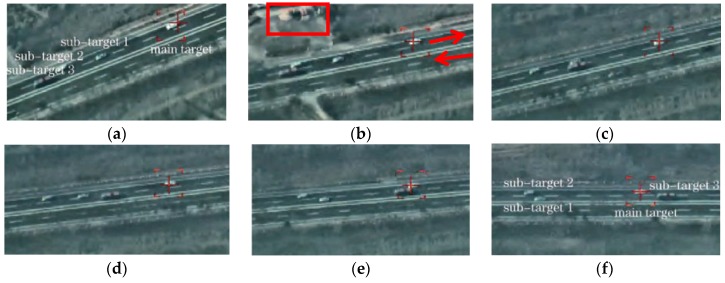
Multiple moving targets in aerial video. (**a**–**f**): frame1, frame 3, frame 4, frame 6, frame 7, frame 9.

This test localizes and tracks four targets moving on a Changji highway in the video taken by the UAV, as shown in Figure 16 (part of the image). The size of every image is 1024 pixels×768 pixels, the pixel size is 5.5 μm×5.5 µm, and the focal length f=73.6 mm. The target in the image center is chosen as main target, and three targets in other positions are chosen as sub-targets. The pixel coordinates of each target in the 1st image and the locations and attitudes data of the electro-optical stabilized imaging system at the corresponding time are shown in Table 8.

**Figure 17:**
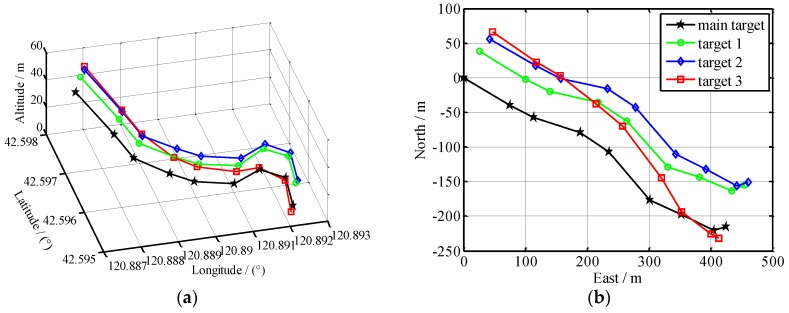
(**a**) The spatial localization of each target; (**b**) The target motion trajectory on the ground.

Nine chronological images are selected from video images to calculate the geo-location data of each target in every image. The resultant spatial position distribution of all the targets is shown in Figure 17a. With the main target position in the 1st image as the origin of coordinates, the spatial positions of all the targets are projected earthwards to obtain their planar motion trails, as shown in Figure 17b. The time span of those nine frames is 30 s.

**Figure 18:**
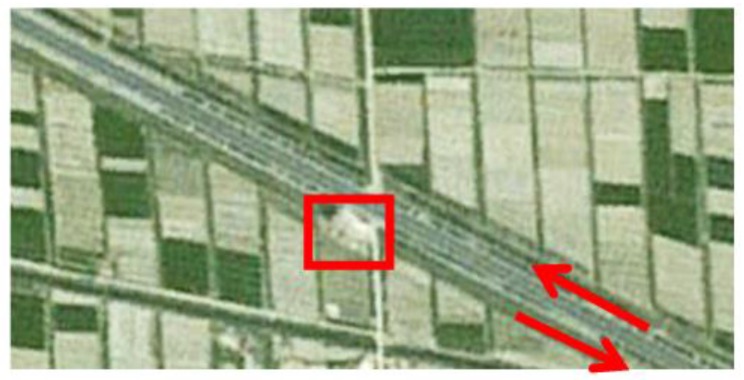
Ortho image of the Changji highway in aerial imagery.

Since both the UAV yaw and the electro-optical stabilized imaging system azimuth are not 0 in general, aerial images have been rotated and distorted somewhat. The aerial orthographic projection of the abovementioned highway, as shown in Figure 18, is acquired from Google Maps. It can be known from Figure 18 that, the actual direction of that highway is northwest–southeast. In China, cars drive on the right side of the road, so the house in Figure 18 is near the highway which is from northwest to southeast direction. In Figure 16b, we can see this house, so all the cars are on the road travelling in a northwest to southeast direction. This coincides with the localization results of Figure 17.

On the basis of Figure 17, the motion of each target has been analyzed as below: the targets in the 1st image, according to their positions from front to back, are sub-target 3, sub-target 2, sub-target 1 and main target in succession; in the 3rd–4th images, the sub-target 2 begins to catch up with and overtake the sub-target 3; in the 4th–6th images, the sub-target 1 begins to catch up with and overtake the sub-target 3; in the 7th–9th images, the main target begins to catch up with and overtake the sub-target 3; at last, all the targets, according to their positions from front to back, are sub-target 2, sub-target 1, main target and sub-target 3 in succession. This coincides completely with the motion law of all the targets in the video image shown in Figure 14, demonstrating that this geo-location algorithm can correctly locate and track multiple moving targets. The speed of each target can also be determined. This test has further verified the correctness of our multi-target geo-location model.

## 6. Conclusions

In order to improve the reconnaissance efficiency of UAV electro-optical stabilized imaging systems, a multi-target localization system based on a UAV electro-optical stabilized imaging system is proposed. First, a target location model and the way to improve the accuracy of multi-target localization are studied. Then, the geodetic coordinates of multiple targets are calculated using homogeneous coordinate transformation. On the basis of this, two methods which can improve the precision of the target localization are proposed: (1) the lens distortion correction method based on the distortion ratio; (2) the RLS filtering method based on UAV dead reckoning. The localization error model is established using Monte Carlo theory. The analysis of the multiple target location algorithm is carried out. The range of the localization error is obtained. In actual flight, the UAV flight altitude is 1140 m. The multi-target localization results are in the range of allowable error. After we use lens distortion correction method in a single image, CEP of the multi-target localization is reduced by 7%. The RLS algorithm can adaptively estimate the location data based on multi-frame images. Compared with multi-target localization based on the single frame image, CEP of the multi-target localization using RLS is reduced by 25%.

The average time to calculate the location data and distortion correction for a single target is 0.4 ms. The normal video rate is 25 fps, so the proposed localization algorithm can locate the 50 targets at the same time in real time. The proposed method significantly reduced the image data processing time, it is convenient to implement the multi-target localization by using other embedded system [42].

However, when a target is out of field of view and re-enters, the operator has to identify the target again. The future research will aim to address this problem. We will try to apply the tracking-learning-detection (TLD) [43] for automatic target detection when a target re-enters the field of view. Due to the difficulty of constructing TLD on the TMS320DM642 (Texas Instruments Incorporated, Dallas, TX, USA), we will leave all these problems for our future research.

## Figures and Tables

**Table 1:** Localization and attitude data of UAV electro-optical stabilized imaging system.

Designation	Symbol	Unit	Nominal Value	Error
UAV longitude	*L*0	°	112.680649	2 × 10^−4^
UAV latitude	*M*0	°	35.125225	1.5 × 10^−4^
UAV altitude	*H*0	m	1140	15
UAV pitch angle	*ε*	°	2.1	0.4
UAV roll angle	*γ*	°	0.0	0.4
UAV yaw angle	*β*	°	290.5	1.5
UAV speed	*V*	m/s	39	0.5
Electro-optical stabilized imaging system’s azimuth angle	*Θ*	°	89.9	0.2
Electro-optical stabilized imaging system’s elevation angle	*Ψ*	°	−112.9	0.2
Laser range finder	*λ*1	m	965	5
Coordinates of sub-target	*(u,v)*	pixel	(386,304), (352,379), (511,277), (379,524) (854,463), (756,584), (685,706)	10
Camera’s focal length	*f*	mm	50.0	0.2

**Table 2:** Errors of multi-target expected location.

	Longitude RMSE/(°)	Latitude RMSE/(°)	Altitude RMSE/m
Main target	0.000307°	0.000166°	25.33 m
Sub-target 1	0.000231°	0.000245°	25.33 m
Sub-target 2	0.000340°	0.000199°	26.12 m
Sub-target 3	0.000292°	0.000265°	25.33 m
Sub-target 4	0.000235°	0.000338°	25.33 m
Sub-target 5	0.000311°	0.000345°	24.56 m
Sub-target 6	0.000532°	0.000166°	26.13 m
Sub-target 7	0.000237°	0.000383°	25.34 m

**Table 3:** Calculated values in the geodesic coordinates of each target.

	Longitude/(°)	Latitude/(°)	Altitude/m
Main target	112.679882	35.125060	171.81
Sub-target 1	112.679058	35.124549	171.82
Sub-target 2	112.680080	35.123993	171.81
Sub-target 3	112.679133	35.125134	171.81
Sub-target 4	112.678723	35.124990	171.82
Sub-target 5	112.680868	35.123948	171.81
Sub-target 6	112.679510	35.123628	171.82
Sub-target 7	112.678275	35.124593	171.82

**Table 4:** Geo-location error of each target in a single image.

	Longitude Error/°/(°)	Latitude Error/°/(°)	Altitude Error/m
Main target	0.000217	0.000027	17.81
Sub-target 1	−0.000081	0.000180	17.82
Sub-target 2	−0.000261	−0.000111	18.81
Sub-target 3	0.000194	0.000207	17.81
Sub-target 4	0.000090	0.000294	17.82
Sub-target 5	−0.000221	−0.000303	16.81
Sub-target 6	−0.000485	−0.000007	18.82
Sub-target 7	−0.000095	0.000344	17.82

**Table 5:** Geo-location errors of multi-target after distortion correction.

	Longitude Error/°	Latitude Error/°	Altitude Error/m	Pixel Coordinates/Pixel
Main target	0.000217°	0.000027°	17.81 m	(512.00, 383.99)
Sub-target 1	−0.000110°	0.000163°	17.82 m	(391.06, 306.08)
Sub-target 2	−0.000259°	−0.000067°	18.81 m	(360.11, 378.32)
Sub-target 3	0.000173°	0.000207°	17.81 m	(511.86, 280.58)
Sub-target 4	0.000016°	0.000285°	17.82 m	(386.18, 516.48)
Sub-target 5	−0.000256°	−0.000243°	16.81 m	(838.99, 458.38)
Sub-target 6	−0.000439°	0.000103°	18.82 m	(746.46, 574.63)
Sub-target 7	−0.000117°	0.000306°	17.82 m	(678.45, 691.36)

**Table 6:** Geo-location accuracy comparison between the proposed algorithm and the algorithms in reference [7].

Algorithm	Error Mean/m	Error Standard/m	Flight Altitude/m
Reference [7]	26.00 ^1^	8.00 ^1^	over 1000 m ^1^
Reference [8]	20.00 ^2^	-	100 m–200 m ^2^
Reference [9]	39.1 ^3^	-	300 m ^3^
Proposed (before correction)	28.98	6.08	1140
Proposed (after correction)	26.91	5.31	1140

^1^ Data from Reference [7]; ^2^ Data from Reference [8]; ^3^ Data from Reference [9].

**Table 7:** Errors of multi-target location after RLS filtering.

	Longitude Error	Latitude Error	Altitude Error
Main target	0.000167°	0.000016°	15.33 m
Sub-target 1	−0.000005°	0.000131°	15.33 m
Sub-target 2	−0.000193°	−0.000086°	16.08 m
Sub-target 3	0.000150°	0.000151°	15.33 m
Sub-target 4	0.000073°	0.000217°	15.33 m
Sub-target 5	−0.000164°	−0.000230°	14.58 m
Sub-target 6	−0.000361°	−0.000007°	16.08 m
Sub-target 7	−0.000065°	0.000255°	15.33 m

**Table 8:** Locations and attitudes data of electro-optical stabilized imaging system.

Designation	Symbol	Unit	Value
UAV longitude	*L*_0_	°	120.906624
UAV latitude	*M*_0_	°	42.608521
UAV altitude	*H*_0_	m	2505
UAV pitch angle	*ε*	°	2.0
UAV roll angle	*γ*	°	−1.8
UAV yaw angle	*β*	°	350.2
Electro-optical stabilized imaging system’s azimuth angle	*Θ*	°	−117.8
Electro-optical stabilized imaging system’s elevation angle	*Ψ*	°	−46.7
Laser range finder	*λ*1	m	3240
Coordinates of sub-target1	(*u*,*v*)	pixel	(453, 342)
Coordinates of sub-target2	(*u*,*v*)	pixel	(476, 251)
Coordinates of sub-target3	(*u*,*v*)	pixel	(504, 213)
Camera’s focal length	*f*	mm	73.6

## References

[1] Deming R.W., Perlovsky L.I. (2007). Concurrent multi-target localization, data association, and navigation for a swarm of flying sensors. Inf. Fusion.

[2] Minaeian S., Liu J., Son Y.-J. (2016). Vision-based target detection and localization via a team of cooperative UAV and UGVs. IEEE Trans. Syst. Man Cybern. Syst..

[3] Morbidi F., Mariottini G.L. (2013). Active target tracking and cooperative localization for teams of aerial vehicles. IEEE Trans. Control Syst. Technol..

[4] Qu Y., Wu J., Zhang Y. Cooperative Localization Based on the Azimuth Angles among Multiple UAVs. Proceedings of the 2013 International Conference on Unmanned Aircraft Systems (ICUAS).

[5] Kwon H., Pack D.J. (2012). A robust mobile target localization method for cooperative unmanned aerial vehicles using sensor fusion qualitye. J. Intell. Robot. Syst. Theory Appl..

[6] Yan M., Du P., Wang H.L., Gao X.J., Zhang Z., Liu D. (2012). Ground multi-target positioning algorithm for airborne optoelectronic system. J. Appl. Opt..

[7] Han K., de Souza G.N. Multiple Targets Geo-Location Using SIFT and Stereo Vision on Airborne Video Sequences. Proceedings of the 2009 IEEE RSJ International Conference on Intelligent Robots and Systems.

[8] Barber D.B., Redding J., McLain T.W., Beard R.W., Taylor C. (2006). Vision-based target geo-location using a fixed-wing miniature air vehicle. J. Intell. Robot. Syst..

[9] Subong S., Bhoram L., Jihoon K. (2008). Vision-based real-time target localization for single-antenna GPS-guided UAV. IEEE Trans. Aerosp. Electron. Syst..

[10] Dobrokhodov V.N., Kaminer I.I., Jones K.D., Ghabcheloo R. Vision-Based Tracking and Motion Estimation for Moving Targets Using Small UAVS. Proceedings of the 2006 American Control Conference.

[11] Yap K.C. (2006). Incorporating Target Mensuration System for Target Motion Estimation Along a Road Using Asynchronous Filter. Master’s Thesis.

[12] Kumar R., Sawhney H., Asmuth J., Pope A., Hsu S. Registration of Video to Geo-Referenced Imagery. Proceedings of the Fourteenth International Conference on Pattern Recognition.

[13] Huang D., Wu Z. The Application of TMS320c64x DSP Assembly Language in Correlation Tracking Algorithms. Proceedings of the 2010 3rd International Congress on Image and Signal.

[14] Zhang Y., Tong X., Yang T., Ma W. (2015). Multi-model estimation based moving object detection for aerial video. Sensors.

[15] Wang Z., Xu J., Huang Z., Zhang X., Xia X.-G., Long T., Bao Q. (2016). Road-aided ground slowly moving target 2D motion estimation for single-channel synthetic aperture radar. Sensors.

[16] Danescu R., Oniga F., Turcu V., Cristea O. (2012). Long baseline stereovision for automatic detection and ranging of moving objects in the night sky. Sensors.

[17] Steen K.A., Villa-Henriksen A., Therkildsen O.R., Green O. (2012). Automatic detection of animals in mowing operations using thermal cameras. Sensors.

[18] Rodriguez-Gomez R., Fernandez-Sanchez E.J., Diaz J., Ros E. (2012). FPGA Implementation for Real-Time Background Subtraction Based on Horprasert Model. Sensors.

[19] Bowring B.R. (1976). Transformation from spatial to geographical coordinates. Surv. Rev..

[20] Li Y., Yun J., Jun W. (2013). Building of rigorous geometric processing model based on line-of-sight vector of ZY-3 imagery. Geomat. Inf. Sci. Wuhan Univ..

[21] Hughes P.C. (2004). Spacecraft Attitude Dynamics.

[22] Fu C., Duan R., Kircali D., Kayacan E. (2016). Onboard robust visual tracking for UAVs using a reliable global-local object model. Sensors.

[23] Liu Z., Wang Z., Xu M. (2016). Cubature information SMC-PHD for multi-target tracking. Sensors.

[24] Perlovsky L.I., Deming R.W. (2013). Maximum likelihood joint tracking and association in strong clutter. Int. J. Adv. Robot. Syst..

[25] Tian W., Wang Y. (2013). Analytic performance prediction of track-to-track association with biased data in multi-sensor multi-target tracking scenarios. Sensors.

[26] Ghirmai T. (2016). Distributed particle filter for target tracking: With reduced sensor communications. Sensors.

[27] Kyristsis S., Antonopoulos A., Chanialakis T., Stefanakis E., Linardos C., Tripolitsiotis A., Partsinevelos P. (2016). Towards autonomous modular UAV missions: The detection, geo-location and landing paradigm. Sensors.

[28] Enayet A., Razzaque M.A., Hassan M.M., Almogren A., Alamri A. (2014). Moving target tracking through distributed clustering in directional sensor networks. Sensors.

[29] Boudriga N., Hamdi M., Iyengar S. (2011). Coverage assessment and target tracking in 3D domains. Sensors.

[30] Dellen B., Erdal Aksoy E., Wörgötter F. (2009). Segment tracking via a spatiotemporal linking process including feedback stabilization in an n-D lattice model. Sensors.

[31] Sun B., Jiang C., Li M. (2016). Fuzzy neural network-based interacting multiple model for multi-node target tracking algorithm. Sensors.

[32] Drap P., Lefèvre J. (2016). An exact formula for calculating inverse radial lens distortions. Sensors.

[33] Wackrow R., Ferreira E., Chandler J., Shiono K. (2015). Camera calibration for water-biota research: The projected area of vegetation. Sensors.

[34] Sanz-Ablanedo E., Rodríguez-Pérez J.R., Armesto J., Taboada M.F.Á. (2010). Geometric stability and lens decentering in compact digital cameras. Sensors.

[35] Bosch J., Gracias N., Ridao P., Ribas D. (2015). Omnidirectional underwater camera design and calibration. Sensors.

[36] Miklavcic S., Cai J. (2013). Automatic curve selection for lens distortion correction using Hough transform energy. IEEE Workshop Appl. Comput. Vis..

[37] Goljan M., Fridrich J. (2014). Estimation of lens distortion correction from single images. Proc. SPIE.

[38] Lee T.Y., Chang T.S., Wei C.H., Lai S.H., Liu K.C., Wu H.S. (2013). Automatic distortion correction of endoscopic images captured with wide-angle zoom lens. IEEE Trans. Biomed. Eng..

[39] Liu J., Sun H., Zhang B., Dai M., Jia P., Shen H., Zhang L. (2007). Target self-determination orientation based on aerial photoelectric imaging platform. Opt. Precis. Eng..

[40] Sheng W., Long Y., Zhou Y. (2011). Analysis of target location accuracy in space-based optical-sensor network. Acta Opt. Sin..

[41] Le Z., Wuzhou L., Yangfeng J. (2013). Localization accuracy evaluation method based on CEP. Command Control Simul..

[42] Kuo D., Gordon D. (2010). Real-time orthorectification by FPGA-based hardware acceleration. Proc. SPIE.

[43] Kalal Z., Mikolajczyk K. (2012). Tracking-Learning-Detection. IEEE Trans. Pattern Anal. Mach. Intell..

